# Self-Selected Versus Standardised Warm-Ups; Physiological Response on 500 m Sprint Kayak Performance

**DOI:** 10.3390/sports8120156

**Published:** 2020-11-30

**Authors:** Amelia F. Dingley, Alexander P. Willmott, John F. T. Fernandes

**Affiliations:** 1 Higher Education Sport, Hartpury University, Gloucester GL19 3BE, UK; Amelia.Dingley@hartpury.ac.uk; 2 School of Sport and Exercise Science, University of Lincoln, Lincoln LN6 7TS, UK; SWillmott@lincoln.ac.uk

**Keywords:** intermittent high-intensity, continuous, kayakers, acute performance, water sport, autoregulation

## Abstract

This study investigated the effectiveness of a self-selected (SS) warm-up on 500 m sprint kayak performance (K500) compared to continuous (CON) and intermittent high intensity (INT)-type warm-ups. Twelve nationally ranked sprint kayakers (age 17.7 ± 2.3 years, mass 69.2 ± 10.8 kg) performed CON (15 min at the power at 2 m·mol^−1^), INT (10 min at 2 m·mol^−1^, followed by 5 × 10 s sprints at 200% power at VO_2max_ with 50 s recovery at 55% power at VO_2max_), and SS (athlete’s normal competition warm-up) warm-ups in a randomised order. After a five-minute passive recovery, K500 performance was determined on a kayak ergometer. Heart rate and blood lactate (BLa) were recorded before and immediately after each warm-up and K500 performance. Ratings of perceived exertion (RPE) were recorded at the end of the warm-up and K500. BLa, heart rate, and RPE were generally higher after the INT than CON and SS warm-ups (*p* < 0.05). No differences in these parameters were found between the conditions for the time trial (*p* > 0.05). RPE and changes in BLa and heart rate after the K500 were comparable. There were no differences in K500 performance after the CON, SS, or INT warm-ups. Applied practitioners can, therefore, attain similar performance independent of warm-up type.

## 1. Introduction

Individual sprint kayaking is contested over 500 m (K500) at both the national and international levels [1]. At the 2019 U23 World Championships, male and female winners completed the event in 107.25 and 116.07 s, respectively [2]. These winning performances were executed by producing and maintaining the highest average boat velocity over the full 500 m [3]. To achieve this, the kayaker must generate and sustain sufficient power output to overcome drag forces that act upon the kayak [4]. When competing in the K500, high level female athletes sustain a power output that is around VO_2peak_ (~45 mL·kg·min^−1^) [5], suggesting the need for a high power output at VO_2peak_. Despite the K500 event being deemed a sprint event, the aerobic energy system contributes ~62% of the energy required [5,6,7]. Moreover, muscular strength and power, maximal aerobic power, and lactate threshold anaerobic capacity have been found to be predictors of sprint kayak performance [6,8,9].

Given the influence of these characteristics (i.e., lactate threshold and maximal aerobic power) on performance, athletes and practitioners have sought to improve those qualities through longitudinal interventions [10,11,12]. For example, Liow and Hopkins [12] reported improvements of 2.3 to 3.4% in sprint performance after six weeks of heavy resistance training (~80% for one repetition maximum). While such an approach can enhance performance, these changes require a prolonged period of time (i.e., weeks). Interventions, such as warm-ups, can enhance performance acutely (i.e., within minutes) [13,14,15,16,17]. Therefore, it is important that practitioners use acute interventions, such as warm-ups, alongside longitudinal interventions to maximise performance.

A warm-up represents a stage of prior physiological or biomechanical activation to enhance athletic performance [18,19,20]. Engaging in a warm-up may improve performance by increasing intramuscular temperature, enhancing ATP resynthesis capacity, improving oxygen (O_2_) kinetics, increasing blood and O_2_ delivery to the muscles, and increasing nerve conduction velocity [13,18,19,20,21]. Consequently, a warm-up can induce a wide range of performance-related enhancements [14,15,22].

Regarding the effects of a warm-up on sprint kayak performance, the literature is limited to two studies [16,21]. Bishop et al. [21] compared three warm-up intensities (15 min continuous at the aerobic threshold (~55% VO_2max_), at the anaerobic threshold (~75% VO_2max_), or midway between the aerobic and anaerobic thresholds (~65% VO_2max_)) on a two-minute all-out kayak ergometer test. The average power in the first half of the test was lower after the anaerobic threshold warm-up than after the lower intensity warm-up [21]. It was suggested that acidemia associated with a more intense warm-up can impair performance [21]. In a subsequent study by Bishop et al. [16], a high-intensity, intermittent warm-up (10 min at 65% VO_2max_ followed by 5 × 10 s sprints at 200% VO_2max_, with 50 s active recovery) was associated with better two-minute all-out sprint kayak performance than a continuous warm-up (15 min at 65% VO_2max_). While these findings are helpful for athletes and coaches, the studies are not without their limitations. In races, kayakers are unlikely to adopt an all-out pacing strategy but instead typically adopt an inverted J pacing strategy [8]. Indeed, Borges et al. [8] noted that the pacing strategy selected by a sprint kayaker is highly variable and dependent on the race level, split distances, and competitive season. Moreover, the two-minute all-out performance used by Bishop et al. [16] might not fully replicate K500 regarding time. Thus, a study that investigates the effects of different warm-ups on K500 performance will increase practitioner confidence when prescribing warm-ups.

Despite the emerging literature on warm-up methods, the efficacy of a self-selected approach has received little attention. Bishop [20] suggests that athletes who perform a warm-up of the same intensity and duration (e.g., a standardised group warm-up) may experience different effects upon performance. Therefore, it could be argued that warm-up duration, intensity, and modality should be selected specifically for each athlete [23]. However, for applied practitioners working with multiple athletes, this approach might not be feasible. It is plausible that experienced athletes, with sufficient training and competition experience, can self-select, and, therefore, individualise their own warm-ups. While the influence of a self-selected strategy on kayak performance has not been explored, limited research has investigated self-selected warm-ups in other modes of exercise (i.e., [17,23]). Athletes who self-selected their warm-ups demonstrated a longer time to exhaustion on the cycle ergometer than those who used no warm-ups or two standardised warm-ups [23]. Some participants did perform better with a standardised warm-up than a self-selected warm-up [23], although this might be due to the heterogenous sample (i.e., the sample consisted of six team sport athletes, two middle-distance runners, and one sprinter). Similarly, swimmers achieved a faster 50 yd freestyle time after performing their own self-selected warm-up than after a set warm-up [17]. Mechanistically, it is plausible that self-selected warm-ups adopted previously [17,23] can improve physiological and psychological preparedness for an athlete. Notwithstanding the mechanistic benefits, which are paramount, if athletes are able to self-select their warm-up effectively, from a practitioner standpoint, this selection will provide an efficient method to enhance performance acutely. However, to date, no study has determined if a self-select warm-up is an effective choice before a K500 time trial. Therefore, the aim of this study was to determine if experienced (i.e., national and international level) sprint kayakers can effectively self-select (SS) their own warm-ups before a K500. To achieve this, we compared a SS warm-up to continuous (CON) and intermittent, high-intensity (INT) warm-ups used previously [16]. Our secondary aim was to report on the internal load experienced (i.e., blood lactate, heart rate, and rating of perceived exertion) during these warm-ups and during the subsequent time trial performance. Given the dearth of comparable studies within sprint kayaking and different SS warm-ups, we propose the null hypothesis for both of our aims: (1) that there will be no differences between performance times and (2) that the internal load will not differ between warm-up modalities.

## 2. Materials and Methods

### 2.1. Participants

Twelve sprint kayak athletes with national and international experience (3 female, 9 male), (age 17.7 ± 2.3 years, body mass 69.2 ± 10.8 kg, stature 176.8 ± 7.4 cm), who were asymptomatic of illness and injury, were recruited by convenience sampling. All athletes were nationally ranked by British Canoeing in sprint categories A-C, where the promotion criterion for the lowest category (C) is completing K500 in sub 132, 118, 144, and 134 s for boys, men, girls, and women, respectively (Table 1). All participants completed a pre-test health questionnaire and written informed consent. Assent was provided by parents/guardians for those under eighteen. Ethical approval was obtained from an institutional ethics committee (Dingley120618).

### 2.2. Experimental Design

**Figure 1:**
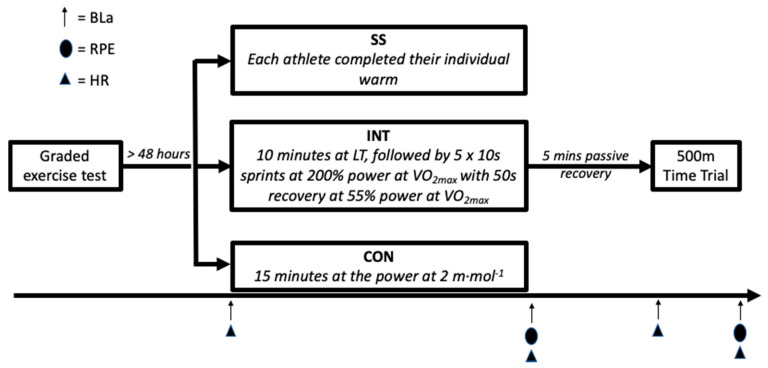
Schematic of the study design. The warms ups were performed in a randomised order. The definitions are as follows: BLa = blood lactate, RPE = rating of perceived exertion, HR = heart rate, LT = lactate threshold, SS = self-selected warm-up, INT = intermittent, high intensity warm-up, CON = continuous warm-up.

The study employed a randomised repeated-measures design in which the participants attended testing on four occasions (Figure 1). Each participant provided details on their SS warm-up prior to taking part in the study. In the initial visit, participants performed a graded exercise test until volitional exhaustion to determine the power at the lactate threshold (LT; i.e., 2 m·mol^−1^) and maximal oxygen uptake (VO_2max_). On subsequent visits, in a randomised order, participants completed a K500 ergometry test that was preceded by either a CON, SS, or INT warm-up. Heart rate (Polar RS400, Kempele, Finland) and capillary blood lactate (BLa; HaB Direct, Warwickshire, UK), from the earlobe, were taken five minutes prior to and immediately after each warm-up. Similarly, the heart rate and BLa were taken immediately before and post the K500. Ratings of perceived exertion (RPE) were measured using the Borg 6–20 scale immediately after each warm-up and K500 performance. Due to technical issues, heart rate data were only collected for eleven participants. Trials were separated by 24 h, and participants were asked to refrain from physical activity for 24 h prior to testing.

### 2.3. Procedures

#### 2.3.1. Lactate Threshold and Maximal Oxygen Uptake

A graded exercise test was performed on a calibrated, wind-braked kayak ergometer (WEBA Sport, Wien, Austria) to determine the power output (W) at LT and VO_2max_ to dictate the warm-up intensities. Participants began at an initial workload of 50 W, with increments of 20 W for males and 15 W for females every four minutes until the athlete reached volitional failure or was unable to maintain the required power output [24]. After each four-minute stage, a one minute passive rest was used for the BLa to be taken [21]. The VO_2max_ was identified by the highest consecutive fifteen seconds [24]. LT was identified at a fixed 2 m·mol^−1^ value [24]. Expired gas samples were collected using a Cosmed K4b2 (Cortex Biophysik, GmbH, Leipzig, Germany). The gas analyser was calibrated prior to each test using a 4.87% CO_2_ and volume O_2_/Nitrogen 16.51/4.87% gas mixture, and the volume sensor was calibrated using a 3 L calibration syringe.

#### 2.3.2. Warm-Up Procedures

- Continuous: Participants performed a 15 min warm-up at the LT. Participants were instructed to maintain a self-selected steady stroke rate (±3 strokes per minute) throughout the warm-up. This warm-up was adopted previously [16,21].
- High-intensity, intermittent: For 10 min, participants performed steady state exercise at the LT. Thereafter, participants completed 5 × 10 s sprints at 200% VO_2max_ with a 50 s active recovery at 55% of the VO_2max_. This warm-up was adopted previously [16,21].
- Self-selected: Participants completed a ~15 min SS warm-up that they would typically complete prior to competition. The full details of these warmups can be found in Table 1.

During the warm-up, the participants were able to see their power and time on the kayak ergometer interfaced with a computer monitor. All warm-ups were followed by a five-minute passive recovery to replicate a competition and allow for sufficient recovery before completing the K500 [25]. In this time, participants remained seated. The athletes did not engage in familiarisation with INT and CON warm-ups; however, all participants were experienced sprint kayak athletes.

### 2.4. Time Trial Performance

The participants completed a 500 m time trial on the same ergometer five-minutes after each warm-up procedure. All participants used a kayak ergometer as part of their training regimes. Participants were asked to start with tension on one blade to replicate the normal starting position on the water and complete the time trial in the shortest time possible. The foot bar position on the ergometer was adjusted to resemble the participant’s own boat set up, and bungee cord tension was adjusted manually to accommodate an increased stroke rate [24,26]. No information was provided regarding the pacing strategy to use. Distance was visible on the kayak ergometer monitor, as the athletes would be aware of this in competition, but not the power output, cadence, or time. Participants were only given their performance times upon completion of the study.

### 2.5. Statistical Analysis

Data were checked for normality and equal variances via Shapiro–Wilk and Levene statistics, respectively, and these assumptions were repeatedly found to be satisfied (*p* > 0.05). A one-way analysis of variance (ANOVA) was used to determine the differences in RPE and time trial performance across warm-up conditions. Separate two-way repeated measure ANOVAs (warm-up type by time) were employed to determine the differences in heart rate and BLa over the warm-up and time trials. When sphericity was violated, the Greenhouse–Geisser correction was used. Where necessary, post hoc t-tests were used to locate differences in specific pairwise comparisons. Between- [(mean change condition/group ‘a’—mean change condition/group ‘b’)/pooled weighted standard deviation of the change score]) and within-condition/group (difference between means/pooled weighted standard deviation) effect sizes (ES; Hedges’ g) [27] and 90% confidence intervals (CI) were calculated to determine the size of the changes between conditions, allowing for a more practical and meaningful explanation of the data. Hedges’ g was used over Cohen’s d, as the former is typically better at reducing bias for smaller sample sizes than the latter. Thresholds for the magnitude of the observed change for each variable were qualified as trivial (<0.20), small (0.20–0.59), moderate (0.60–1.19), large (1.20–1.99), or very large (>2.00) [28]. The alpha was set at 0.05.

## 3. Results

### 3.1. Internal Load during the Warm-Up

Comparing the values pre and post warm-up, a two-way repeated measures ANOVA revealed effects for warm-up (F = 8.0, *p* = 0.003), time (F = 18.6, *p* = 0.001) and warm-up by time (F = 6.5, *p* = 0.006) on BLa (Table 2). The post hoc analysis demonstrated significant increases in BLa after the warm-up for the CON (t = −5.1, *p* < 0.001) and INT (t = −4.5, *p* = 0.001) but not SS (t = −1.7, *p* = 0.120). Notably, moderately greater increases in BLa were observed after the INT versus SS (ES = −0.91 ± 0.71) and CON (ES = −0.73 ± 0.71) warm-ups. Differences in BLa changes between CON and SS conditions were trivial (ES = 0.19 ± 0.67).

The effects of the warm-up (F = 0.7, *p* = 0.504) and warm-up by time (F = 2.0, *p* = 0.174) were non-significant for heart rate for the same period, although there was a main effect for time (F = 84.7, *p* < 0.001). Increases in heart rate after the warm-up were significant for CON (t = −7.2, *p* = 0.001), SS (t = −5.3, *p* = 0.001) and INT (t = −8.6, *p* = 0.001) conditions. The differences in changes in heart rate between the conditions were trivial for the CON versus INT (ES = 0.16 ± 0.69) and moderate for the CON versus SS and INT versus SS (ES = 0.74 ± 0.74, ES = 0.67 ± 0.72) comparisons.

**Figure 2:**
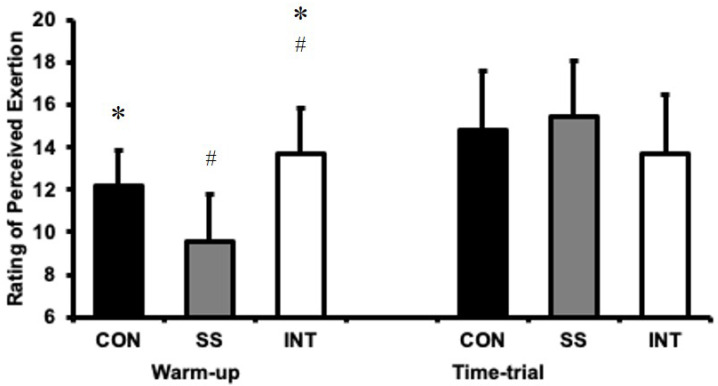
Ratings of perceived exertion (mean ± SD) after the warm-up and time-trial for continuous (CON), self-selected (SS), and intermittent, high-intensity (INT) conditions. * denotes a significant difference from SS (*p* < 0.05). # denotes a significance difference from CON (*p* < 0.05).

A one-way ANOVA identified differences in RPE after the warm-up between conditions (F = 13.2, *p* < 0.001; Figure 2, whereby the differences were significant for all comparisons. Notably, RPE was higher after INT than CON (t = −2.3, *p* = 0.04, ES = −0.65 ± 0.69) and SS (t = −4.5, *p* = 0.001, ES = 1.78 ± 0.79), and CON was higher than SS (t = 3.1, *p* = 0.01, ES = 1.13 ± 0.73).

### 3.2. Time Trial Performance

**Figure 3:**
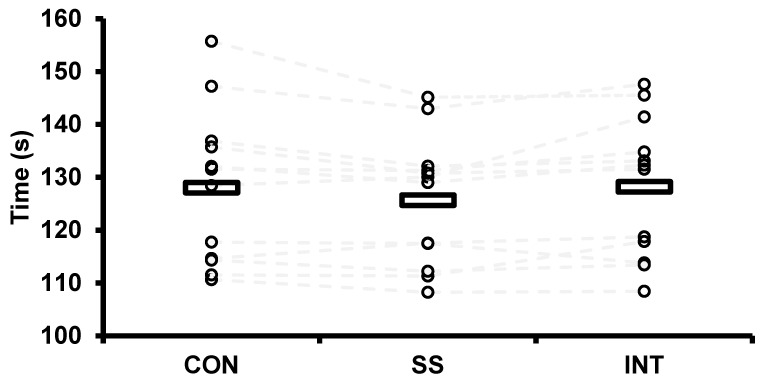
Sprint kayak time trial performance after continuous (CON), self-selected (SS), and intermittent, high-intensity (INT) warm-ups. For comparative purposes, grey lines connect the individual times for each participant. The black rectangles denote the average of each condition.

Figure 3 displays the mean and individual values for the time trial after each warm-up. There were no differences in time trial performance (128.0 ± 14.6, 125.7 ± 11.7 and 128.2 ± 13.3 s for CON, SS and INT, respectively) between conditions (F = 2.3, *p* = 0.150). The magnitude of the differences between all comparisons was trivial (ES = 0.19 ± 0.67, 0.19 ± 0.67 and 0.02 ± 0.67 for CON vs. SS, SS vs. INT and CON vs. INT, respectively).

### 3.3. Internal Load during the Time Trial

There was a main effect of time and time trial by time on BLa (F = 70.1, *p* < 0.001 and F = 3.9, *p* = 0.035, respectively) and heart rate (F = 31.1, *p* < 0.001 and F = 5.5, *p* = 0.012, respectively; Table 3). A paired sample *t*-test demonstrated increases in the BLa and heart rate for CON (t = −5.9, *p* < 0.001 and t = −4.2, *p* = 0.002, respectively), SS (t = −8.6, *p* < 0.001 and t = −4.7, *p* = 0.001, respectively), and INT (t = −6.4, *p* < 0.001 and t = −3.9, *p* = 0.002, respectively). The differences in changes in BLa were moderate for the CON and SS (ES = 0.59 ± 0.68) and SS and INT (ES = 0.83 ± 0.70) comparisons but trivial for CON versus INT (ES = 0.19 ± 0.67). Post hoc analysis demonstrated a significant increase in heart rate from pre- to post-time trial in the CON (t = −4.2, *p* = 0.002), SS (t = −4.7, *p* = 0.001), and INT (t = −3.9, *p* = 0.002) warm-ups. The INT versus SS (ES = 0.67 ± 0.72) and CON versus SS (ES = 0.59 ± 0.68) heart rates showed differences that were small–moderate in their change scores. The comparison between CON and INT revealed trivial differences (ES = 0.19 ± 0.67).

The one-way ANOVA revealed no differences between the post time trial RPEs for each condition (*p* = 0.171). Indeed, the magnitude of the effects between CON versus SS, SS versus INT, and CON versus INT were small (ES = 0.22 ± 0.67, 0.59 ± 0.68, and 0.38 ± 0.68, respectively).

## 4. Discussion

This study compared the effectiveness of a self-selected warm-up on 500 m sprint kayak performance among experienced athletes with that of two standardised warm-ups. The K500 time trial performance after a SS warm-up was not different to that after a CON or INT warm-up. Moreover, the changes in internal load markers (blood lactate, heart rate, and RPE) were similar after the time trial performance. Therefore, sprint kayakers competing in the K500 can attain similar performance with a variety of warm-ups. Practically, coaches may wish for athletes to self-select their own warm-ups, as this is likely easier to manage.

The CON and INT warm-ups resulted in greater increases in BLa than the SS warm-up. These findings are supported by previous observations of increased BLa after CON- and INT-type warm-ups [16,21]. Like these reports, the increase in BLa was greater for those warm-ups performed at a higher intensity (i.e., INT > CON). To our knowledge, no study has examined changes in BLa after an SS warm up. Nonetheless, the higher BLa data indicate that the glycolytic pathway was used to a greater extent during the INT and CON conditions than under SS conditions. The elevations in heart rate over the course of the warm-ups were generally similar between conditions. This is in contrast to the results of Bishop and colleagues [21], who reported increases in heart rate that followed the intensity of the warm-up; a higher intensity warm-up raised the heart rate to a greater extent. Similarly, Balilionis et al. [17] found that an SS swim warm-up resulted in greater increases in heart rate than a standardised warm-up (50 yards at 40% of the swimmers’ maximal effort and 50 yards at 90%). However, our data are comparable to those of Mandengue et al. [23], who noted similar changes in heart rate irrespective of warm-up intensity. These comparable changes in heart rate in our study suggest similar alterations in cardiac sympathetic and parasympathetic activity, which aim to deliver oxygen to the working musculature [29]. Like BLa, athletes perceived exertion followed a similar pattern, i.e., INT > CON > SS. Balilionis et al. [17] reported higher RPE after an SS warm-up than a short standardised warm-up. Notably, the standardised warm-up provided by Balilionis and colleagues [17] consisted of only 90 m swimming, whereas participants in the SS warm-up typically performed over ~1300 m. Nonetheless, the differences in RPE between conditions suggest different levels of motor command to the working muscles across the different warm-ups [30]. Collectively, these internal load data in this investigation suggest that the internal load for the INT warm-up was greater than that of the CON and SS warm-ups.

This is the first study to investigate the effects of SS warm-ups on sprint kayak performance. Despite differences in the intensity (as noted by the differences in the internal load) and types, the sprint kayak performance was similar under all the warm-up conditions. Our data are in contrast to those of Bishop and colleagues [16], who observed enhanced power outputs during a 2 min all-out sprint kayak test after an INT warm-up compared to a CON one. The reason for the differences between our findings and those of Bishop et al. [16] are unclear but might reflect differences in the nature of an all-out test versus a time trial. Specifically, the pacing strategies of sprint kayakers are highly variable during a time trial and likely reflect an inverted-J strategy rather than an all-out one [8]. Nonetheless, the changes in BLa and heart rate were greater in the SS condition than CON and INT, but this was not reflected in the RPE scores, which were comparable between conditions. That the internal load was generally lower during the WU but greater after the time trial in the SS condition than under CON and INT suggests that when self-selecting their warm-ups, sprint kayakers ‘paced/spared’ their aerobic or anaerobic capacities [16,19,20]. However, this potential pacing/sparing was not manifested as improved time trial performance in this study. Holistically, these data suggest that experienced sprint kayakers can perform the K500 using a variety of warm-up strategies. However, given the potential psychological preparedness that a SS warm-up might have [19,25], practitioners may wish to prescribe this warm-up.

This study was performed in a laboratory setting on a kayak ergometer and did not fully mirror the demands of on-water paddling but did simulate the physiological demands of short-term, high intensity kayaking; this choice, however, removed confounding factors such as differences in wind and temperature between days [31]. Moreover, we did not include a condition without a warm-up, so our data compared warm-ups rather than investigating their performance-enhancing effects per se. Nonetheless, from our perspective, it is highly unlikely that a national or international level athlete will compete without performing a warm-up. Readers should be aware that while we included nationally and internationally ranked kayakers, our sample was not homogenous in the sense that the sample features a mixed sex and included a variety of ages. However, our data still provide novel insights into the use of self-selected warm-ups for sprint kayak warm ups among high level athletes. Future work may wish to address these limitations.

## 5. Conclusions

Sprint kayak performance was not significantly different after CON, SS, and INT type warm-ups. Although there was some evidence (i.e., the attenuated internal load) that participants ‘paced/preserved’ their capacity more during the SS warm-up compared to the CON and INT warm-ups, this did not manifest as enhanced performance. Applied practitioners may, therefore, prescribe a variety of warm-ups before sprint kayak performance. Practically, however, it might be more viable for athletes to perform their own self-selected warm-ups.

## Figures and Tables

**Table 1:** Characteristics and **i**ndividual warm-ups for each participant. PM denotes perceived maximum effort.

Participant	Ranking	Age	Mass (kg)	Stature (cm)	VO_2max_ (mL·kg·min^−1^)	Warm up
1	Men’s B	21	79	181	42	500 m relaxed paddle, 3–4 × 20 stroke efforts
2	Women’s B	18	75	186	32	250 m relaxed paddle, power strokes 250 m, 3–5 × 30 stroke build up (10, 10, 10), 250m paddle, 1 × 30 stroke rolling start, 1 × 30 strokes standing start
3	Boys B	15	56	179	39	500 m relaxed paddle, 2 × standing starts, 2 × 40 stroke build-up of power same stroke rate upping every 10 until max on 40, 2 × standing starts.
4	Boys C	15	72	181	43	750 m paddle with power strokes on/off, 200 m paddle, 3 × 30 stroke starts.
5	Men’s B	20	76	180	39	90 s relaxed paddle, 3 × 30 at 70% PM rolling starts, 3 × 20 at 80% PM rolling starts, 2 × 20 at 90% PM, 1 rolling start and 1 standing start, 1 × 15 at 100% PM standing start.
6	Boys B	16	77	182	55	3 × 30 strokes at 70% PM, 2 × 20 strokes at 80% PM, 1 × 20 strokes at 90% PM, 1 × 20 strokes at 95% PM.
7	Boys A	16	51	165	65	500 m relaxed paddle, 3 × 30 strokes at 70% PM, 2 × 20 strokes at 80% PM, 2 × 20 strokes at 90% PM, 1 × 20 strokes at 100% PM, 2 build ups (10 strokes 70% PM, 10 strokes 80% PM, 10 strokes 90% PM, 5 strokes at 100% PM.
8	Men’s A	20	86	183	46	500 m relaxed paddle, 3 × 30 strokes at 70% PM, 20 strokes at 80% PM, 20 strokes at 80–90% PM, 10 strokes at 60% PM.
9	Girls A	18	59	160	36	500 m perceived LT threshold paddle, 3 × 30 strokes at 70% PM, 2 × 20 strokes at 80% PM, 1 × 20 strokes at 90% PM, 2–3 standing starts × 40 strokes.
10	Men’s A	21	79	179	50	3 × 30 strokes at 70% PM, 2 × 20 strokes at 80% PM, 1 × 20 strokes at 90% PM, 1 × 30 strokes at 70% PM, 1 × 20 strokes at 95% PM, 1 × 30 strokes at 70% PM.
11	Girls C	15	61	171	38	2 × 30 strokes at 70% PM, 2 × 25 strokes at 80% PM, 2 × 15 strokes at 95% PM, 2 × standing starts.
12	Boys C	17	59	174	48	500 m relaxed paddle, 3 × 30 strokes at 70% PM, 2 × 20 strokes at 80% PM, 2 × 20 strokes at 90% PM, 1 × 20 strokes at 100% PM, 2 build ups (10 strokes at 70% PM, 10 strokes at 80% PM, 10 strokes at 90% PM, 5 strokes at 100% PM.

**Table 2:** Blood lactate and heart rate values (mean ± SD) pre and post warm-up for continuous (CON), self-selected (SS) and intermittent, high-intensity (INT) conditions. * denotes a significant different from pre to post within-conditions (*p* < 0.05).

		*Pre*	*Post*
**CON**	***BLa (mmol·L^−1^)***	1.1 ± 0.2	2.0 ± 0.6 *
	***Heart rate (BPM)***	63.7 ± 3.5	108.6 ± 18.8 *
**SS**	***BLa (mmol·L^−1^)***	1.1 ± 0.2	1.8 ± 1.3
	***Heart rate (BPM)***	66.1 ± 12.0	96.7 ± 9.4 *
**INT**	***BLa (mmol·L^−1^)***	1.1 ± 0.3	3.3 ± 1.6 *
	***Heart rate (BPM)***	67.2 ± 8.8	107.7 ± 18.2 *

**Table 3:** Blood lactate and heart rate values (mean ± SD) pre- and post-time trial for continuous (CON), self-selected (SS), and intermittent, high-intensity (INT) conditions. * denotes a significant different from the pre to post within-condition (*p* < 0.05).

		*Pre*	*Post*
**CON**	***BLa (mmol·L^−1^)***	1.9 ± 0.5	6.0 ± 2.1 *
	***Heart rate (BPM)***	102.4 ± 16.1	131.2 ±19.1 *
**SS**	***BLa (mmol·L^−1^)***	1.7 ± 1.4	7.1 ± 2.3 *
	***Heart rate (BPM)***	95.8 ± 13.1	136.4 ± 19.4 *
**INT**	***BLa (mmol·L^−1^)***	3.2 ± 1.6	6.9 ± 2.3 *
	***Heart rate (BPM)***	100.4 ± 21.5	122.1 ± 20.7 *

## References

[1] Bishop D., Bonetti D., Dawson B. (2002). The influence of pacing strategy on VO2 and supramaximal kayak performance. Med. Sci. Sports Exerc..

[2] ICF 2019 ICF Canoe Sprint World Chamionships. https://www.canoeicf.com/canoe-sprint-world-championships/szeged-2019.

[3] Baker J. Biomechanics of paddling. Proceedings of the 30th Conference of the International Society of Biomechanics in Sports (ISBS).

[4] Baudouin A., Hawkins D. (2002). A biomechanical review of factors affecting rowing performance: Commentary. Br. J. Sports Med..

[5] Bishop D. (2000). Physiological predictors of flat-water kayak performance in women. Eur. J. Appl. Physiol..

[6] van Someren K.A., Howatson G. (2008). Prediction of flatwater kayaking performance. Int. J. Sports Physiol. Perform..

[7] Zouhal H., Le Douairon Lahaye S., Ben Abderrahaman A., Minter G., Herbez R., Castagna C. (2012). Energy system contribution to Olympic distances in flat water kayaking (500 and 1000 m) in highly trained subjects. J. Strength Cond. Res..

[8] Borges T.O., Bullock N., Coutts A.J. (2013). Pacing characteristics of international sprint kayak athletes. Int. J. Perform. Anal. Sport.

[9] McKean M.R., Burkett B.J. (2014). The influence of upper-body strength on flat-water sprint kayak performance in elite athletes. Int. J. Sports Physiol. Perform..

[10] Aitken D.A., Jenkins D.G. (1998). Anthropometric-based selection and sprint kayak training in children. J. Sports Sci..

[11] Bonetti D.L., Hopkins W.G., Kilding A.E. (2006). High-intensity kayak performance after adaptation to intermittent hypoxia. Int. J. Sports Physiol. Perform..

[12] Liow D.K., Hopkins W.G. (2003). Velocity specificity of weight training for kayak sprint performance. Med. Sci. Sports Exerc..

[13] Kilduff L.P., Finn C.V., Baker J.S., Cook C.J., West D.J. (2013). Preconditioning strategies to enhance physical performance on the day of competition. Int. J. Sports Physiol. Perform..

[14] Burnley M., Doust J.H., Jones A.M. (2005). Effects of prior warm-up regime on severe-intensity cycling performance. Med. Sci. Sports Exerc..

[15] Stewart I.B., Sleivert G.G. (1998). The effect of warm-up intensity on range of motion and anaerobic performance. J. Orthop. Sports Phys. Ther..

[16] Bishop D., Bonetti D., Spencer M. (2003). The effect of an intermittent, high-intensity warm-up on supramaximal kayak ergometer performance. J. Sports Sci..

[17] Balilionis G., Nepocatych S., Ellis C.M., Richardson M.T., Neggers Y.H., Bishop P.A. (2012). Effects of different types of warm-up on swimming performance, reaction time, and dive distance. J. Strength Cond. Res..

[18] Fairbank M., Highton J., Twist C. (2019). Passive heat maintenance after an initial warm-up improves high-intensity activity during an interchange rugby league movement simulation protocol. J. Strength Cond. Res..

[19] Bishop D. (2003). Warm up I: Potential mechanisms and the effects of passive warm up on exercise performance. Sports Med..

[20] Bishop D. (2003). Warm up II: Performance changes following active warm up and how to structure the warm up. Sports Med..

[21] Bishop D., Bonetti D., Dawson B. (2001). The effect of three different warm-up intensities on kayak ergometer performance. Med. Sci. Sports Exerc..

[22] Burkett L.N., Phillips W.T., Ziuraitis J. (2005). The best warm-up for the vertical jump in college-age athletic men. J. Strength Cond. Res..

[23] Mandengue S.H., Seck D., Bishop D., Cissé F., Tsala-Mbala P., Ahmaidi S. (2005). Are athletes able to self-select their optimal warm up?. J. Sci. Med. Sport.

[24] Tanner R., Gore C.J. (2013). Physiological Tests for Elite Athletes.

[25] Alanazi H.M. (2016). Role of warming-up in promoting athletes health and skills. Int. J. Sci. Res. Publ..

[26] Lok L.Y. Biomechanics study in sprint kayaking using simulator and on-water measurement instrumentation: An overview. Proceedings of the 3rd Malaysian Postgraduate Conference.

[27] Dankel S.J., Mouser J.G., Mattocks K.T., Counts B.R., Jessee M.B., Buckner S.L., Loprinzi P.D., Loenneke J.P. (2017). The widespread misuse of effect sizes. J. Sci. Med. Sport.

[28] Hopkins W.G., Marshall S.W., Batterham A.M., Hanin J. (2009). Progressive statistics for studies in sports medicine and exercise science. Med. Sci. Sports Exerc..

[29] Rezk C.C., Marrache R.C.B., Tinucci T., Mion D., Forjaz C.L.M. (2006). Post-resistance exercise hypotension, hemodynamics, and heart rate variability: Influence of exercise intensity. Eur. J. Appl. Physiol..

[30] de Morree H.M., Klein C., Marcora S.M. (2012). Perception of effort reflects central motor command during movement execution. Psychophysiology.

[31] van Someren K.A., Phillips G.R., Palmer G.S. (2000). Comparison of physiological responses to open water kayaking and kayak ergometry. Int. J. Sports Med..

